# Neurodevelopmental conditions and adaptive functioning – a co‐twin control study

**DOI:** 10.1111/jcpp.70073

**Published:** 2025-11-04

**Authors:** Johan Isaksson, Filippa Eklund, Karl Lundin Remnélius, Melissa H. Black, Sven Bölte

**Affiliations:** ^1^ Child and Adolescent Psychiatry Unit, Department of Medical Sciences Uppsala University Uppsala Sweden; ^2^ Center of Neurodevelopmental Disorders (KIND), Department of Women's and Children's Health Karolinska Institutet & Centre for Psychiatry Research, Stockholm Health Care Services, Region Stockholm Stockholm Sweden; ^3^ Department of Community and Clinical Health School of Allied Health, Human Services & Sport, La Trobe University Melbourne Australia; ^4^ Curtin Autism Research Group, Curtin School of Allied Health Curtin University Perth WA Australia; ^5^ Child and Adolescent Psychiatry, Stockholm Health Care Services, Region Stockholm Stockholm Sweden

**Keywords:** Adaptive behavior, impairment, ADHD, ASD, intellectual disability

## Abstract

**Background:**

Challenges in adaptive or daily functioning are inherent to diagnostic criteria for neurodevelopmental conditions (NDCs). However, less is known regarding the influence of factors that may confound the association between NDCs and adaptive functioning. Therefore, we examined the associations between different NDCs and adaptive functioning while adjusting for co‐occurring conditions, genetics, and shared environment.

**Methods:**

We used a co‐twin control study design in a sample of Swedish twins (*N* = 314, age range 8–21 years), including both monozygotic (MZ) and dizygotic (DZ) twins. Adaptive function was assessed using the parent‐rated Adaptive Behavior Assessment System, second edition. A generalized estimating equations (GEE) model was fitted, using NDC diagnoses of Autism Spectrum Disorder, Attention‐Deficit/Hyperactivity Disorder (ADHD), and Intellectual Disability (ID), as well as other psychiatric conditions, as exposure and adaptive functioning as the outcome. The model was first fitted across twin pairs and subsequently within the twin pairs, thus adjusting for genetic and shared environmental influences. Interaction effects of age and sex by different NDCs on adaptive functioning were assessed.

**Results:**

All forms of NDCs were independently associated with challenges in adaptive function across pairs. The co‐occurrence of multiple NDCs was associated with adaptive functioning, with a greater number of NDCs being associated with more functioning challenges. Higher age was associated with more challenges in adaptive functioning among autistic individuals. In the within‐pair models, the association remained for autism and ID, but the association between ADHD and adaptive functioning was lost in the MZ sub‐sample when adjusting fully for all genetic factors.

**Conclusions:**

NDCs are associated with challenges in adaptive function, even when adjusting for other psychiatric conditions, stressing the importance of adequate community support. Findings indicate the importance of non‐shared environmental factors for understanding the challenges in adaptive function experienced by individuals with autism and ID and genetic factors for individuals with ADHD.

## Background

Neurodevelopmental conditions (NDCs) are expressions of divergent structural and functional maturation of the central nervous system, associated with challenges in navigating everyday life demands (Thapar, Cooper, & Rutter, 2017). Some of the most common NDCs are Attention‐Deficit/Hyperactivity Disorder (ADHD), Autism Spectrum Disorder (from hereafter, autism), and Intellectual Disability (ID). These conditions are generally understood as the extreme end of a quantitative distribution of NDC traits in the general population (Colvert et al., 2015; McLennan, 2016). Though prevalence rates are difficult to estimate, the occurrence of ADHD has been suggested to be between 5% and 11%, autism between 0.7% and 3%, and up to 1% for ID (Francés et al., 2022; Morinaga et al., 2024). DSM‐5 (Diagnostic and Statistical Manual of Mental Disorders (DSM), APA, 2013) and ICD‐11 (WHO, 2019) behaviorally define ADHD by patterns of inattention and/or hyperactivity‐impulsivity, autism by challenges in social communication and interaction alongside restricted repetitive behavior, and ID by cognitive ability two standard deviations below the population mean. Importantly, to qualify for a clinical diagnosis, these behaviors must impact adaptive or daily functioning. Although functional impairment is a diagnostic criterion for NDC diagnoses, the construct of functioning is only crudely described in the diagnostic manuals. However, functioning is essential to capture because it informs how a person is *living* with a condition and indicates support needs and future outcomes (Forbes et al., 2023).

Adaptive or daily functioning is a construct referring to skills that reflect the individual's independent capacities to meet personal, social, and practical demands in day‐to‐day situations. These skills usually encompass conceptual, social, and practical domains, and the impact on functioning in specific areas may differ depending on different NDCs. In a recent review on functional impairments in ADHD (Kosheleff, Mason, Jain, Koch, & Rubin, 2023), ADHD was associated with challenges across various areas of everyday life. In the social domain, individuals with ADHD could experience difficulties with romantic and peer relationships. In the conceptual domain, they had challenges with educational achievement, while in the practical domain, they could experience more employment difficulties, accidents, and criminal activity. For autism, the social domain is particularly relevant, with challenges in socialization skills being emphasized (Saulnier, Klaiman, & McQueen, 2022), although difficulties in the conceptual and practical domains have also been reported (Laghi et al., 2022). Similarly, in ID, challenges tend to occur broadly across social, conceptual, and practical domains (Patel, Cabral, Ho, & Merrick, 2020).

NDCs often co‐occur. For children and adolescents with autism, ADHD and ID are the most common co‐occurring conditions, with approximately one‐quarter of the autistic population also diagnosed with ADHD or ID (Mutluer et al., 2022). Similar numbers have been reported for individuals with ADHD, where approximately 11%–30% meet the criteria for autism using standardized diagnostic tools (Antshel & Russo, 2019), although estimates may differ depending on how autism is measured and across clinical and community samples. As the co‐occurrence of NDCs is common, it is important to assess both the influence on adaptive or daily functioning for single NDCs and their cumulative impact. For instance, in a sample of autistic individuals, ADHD symptoms predict challenges in adaptive behavior above autism (Yerys, Bertollo, Pandey, Guy, & Schultz, 2019), while for autistic children and adolescents without ID, IQ was only a weak predictor of adaptive functioning and, as such, is an imprecise proxy for functional ability in autism (Alvares et al., 2020). The presence of other NDCs is, however, seldom adjusted for when assessing the impact on adaptive or daily functioning. Some studies have compared groups of individuals with different co‐occurring conditions, finding that children diagnosed with autism and co‐occurring ADHD or clinically significant ADHD symptoms have greater challenges in adaptive functioning (Ashwood et al., 2015; Liu et al., 2021; Sikora, Vora, Coury, & Rosenberg, 2012; Ward et al., 2022), as well as a poorer health‐related quality of life (Sikora et al., 2012) compared to autistic children without ADHD symptoms, indicating that concurrent ADHD may add to a potential cumulative impact on adaptive functioning. Also rarely accounted for when examining adaptive or daily functioning in NDCs is the presence of psychiatric conditions such as anxiety and depression, despite them contributing to impairment (Scott et al., 2014) and frequently co‐occurring in NDC populations. A recent study in child and adolescent mental health services found that 26.5% of children with ADHD and 36% of autistic children had an anxiety disorder, while for depressive disorder, 5% of those with ADHD and 8% of autistic children met criteria (Hansen, Oerbeck, Skirbekk, Petrovski, & Kristensen, 2018).

Both sex and age are important to consider in the context of functioning and NDCs. NDCs are more commonly diagnosed in males compared to females and are usually diagnosed in childhood, being more common in younger populations (Thapar et al., 2017). Tillmann et al. (2019) found that older age, but not sex, negatively impacted adaptive functioning in autistic individuals, although there was a large variation in adaptive functioning scores across all ages. A further study found that autistic boys performed better than autistic girls in social adaptive functioning at older ages, while the opposite was found for ADHD, where girls with ADHD scored higher on social skills than males across all ages (Mahendiran et al., 2019). Although there appear to be sex and age influences on adaptive functioning, examination of a broader range of co‐occurring conditions, including ID, has seldom been considered when investigating any sex or age specificity in the association between NDCs and adaptive functioning.

Since NDCs are regarded as highly heritable conditions (Faraone et al., 2021; Lichtenstein et al., 2022; Tick, Bolton, Happé, Rutter, & Rijsdijk, 2016), familial factors are likely to influence adaptive or daily functioning in individuals with NDCs. This includes not only shared genetics but also shared non‐genetic factors. Socioeconomic status (SES), for example, has been found to account for variability in adaptive functioning in autistic preschool‐aged children (Hodge et al., 2021), and parenting style may be related to the child's adaptive functioning (Carroll, 2022). A family history of depression or shyness has been found to be associated with variation in adaptive behaviors, especially for socialization scores, in autistic individuals (Mazefsky, Williams, & Minshew, 2008). These findings highlight the importance of disentangling genetic and environmental contributions to these outcomes, as both genetic (e.g. a unidirectional genetic susceptibility to poor socialization skills conferred by familial depression) and contextual factors (e.g. modeling and general family functioning) may drive the link between family history factors and adaptive behavior (Mazefsky et al., 2008). Twin and family studies that can differentiate genetic and environmental contributions while implicitly controlling for a large number of confounding familial factors may prove particularly powerful in exploring the impact of broad influences on adaptive or daily functioning in NDCs, but these study designs are scarce in this field. As Mazefsky et al. (2008) highlight in their study, clarifying the relative roles of heritable and contextual factors is essential for understanding why adaptive behavior varies across individuals with similar neurodevelopmental diagnoses. Co‐twin control studies can be an especially important tool for investigating causal contributions, as the methodology automatically controls for all shared environments (including socioeconomic background and parenting styles) between the twins, as well as genetic factors, with dizygotic (DZ) twins sharing approximately 50% of their genome and monozygotic (MZ) twins sharing 100% of their genome (Vitaro et al., 2009). Hence, if the association between NDCs and adaptive functioning remains within twin pairs, this indicates a non‐shared contribution (i.e. factors specific for each twin are indicated to be directly involved in the pathway of adaptive functioning).

One study, including 204 families, found that autistic siblings were more similar on measures of adaptive functioning than unrelated autistic children, with twin correlations indicating strong genetic effects for some skill domains and the influence of shared environmental factors for others (Goin‐Kochel, Mazefsky, & Riley, 2008). Another study of young adult twins, including pairs diagnosed with ADHD and autism, reported a large and significant genetic overlap between ADHD and self‐reported functional impairment across multiple domains, whereas autism was associated both phenotypically and genetically with difficulties in social adaptive functioning (Aydin et al., 2022). Even though no co‐twin control design was applied, which would enable an adjustment for familial confounding to determine the contribution of specific NDCs to adaptive functioning over and beyond familial factors, these results indicate that shared genetic factors may explain the association between ADHD and challenges in functioning.

To summarize, even though NDCs are defined by challenges in adaptive or daily functioning, the contribution of specific NDCs when considering co‐occurring conditions and familial factors remains to be determined. Addressing this research question is important for understanding the etiology of adaptive functioning and whether challenges in functioning are more context‐dependent. By disentangling the relationship between specific NDCs and functioning from familial confounding, we can better determine the focus of clinical interventions to mitigate challenges in functioning. By applying a co‐twin control design in a twin cohort enriched for NDCs, we saw the opportunity to address this research question. There is, to our knowledge, no other study examining the link between NDCs and adaptive function using a co‐twin control design. The aim of the present study was to examine (a) the association between different NDCs and adaptive functioning and if associations between NDCs and functioning remain after adjusting for co‐occurring conditions, (b) if age and sex influence the association between NDCs and adaptive functioning, (c) if the co‐occurrence of multiple NDCs is associated with increasing challenges in adaptive functioning, and (d), and if the associations between NDCs and adaptive functioning remain within pairs when adjusting for genetics and shared environment. We hypothesize that (a) all forms of NDCs are associated with challenges in adaptive functioning and that these associations will remain when adjusting for other NDCs and psychiatric conditions, (b) that age and sex modulate the relationship between NDCs and adaptive function, (c) that co‐occurring NDCs are associated with more challenges in adaptive functioning and that (d) associations between NDCs and challenges in adaptive function are attenuated when adjusting for genetics and shared environment given the high heritability of NDCs, but also that the association remains beyond this familial confounding indicating a non‐shared contribution of NDCs on adaptive functioning since impaired functioning is a diagnostic criterion in NDCs.

## Methods

### Procedure

The study used data from the Roots of Autism and ADHD Twin Study in Sweden (RATSS), which includes comprehensively clinically phenotyped twin pairs from all over Sweden, comprising both typically developing twins and twin pairs with at least one twin diagnosed with a NDC. Twins in RATSS are mainly recruited from the population‐based Child and Adolescent Twin Study in Sweden (CATSS) and the Young Adult Twins in Sweden Study (YATSS) (Bölte et al., 2014; Isaksson et al., 2022). Zygosity was determined by single nucleotide polymorphism markers (Hannelius et al., 2007) and, in a few cases (*n* = 17), by physical appearance using a 4‐item questionnaire. The study was approved by the Swedish Ethical Review Authority, and written and oral informed consent was obtained from all participants.

### Participants

The RATSS sample includes 468 participants. For the present study we excluded all participants 22 years or older (*n* = 117), as we used parent‐rated ABAS‐II with established norms up to the age of 21; those where the co‐twin had a different sex (*n* = 19); one participant who requested that the data be removed from RATSS; and those missing data on the ABAS‐II (*n* = 17). We evaluated differences between participants with missing ABAS‐II data and those with complete data on a set of demographic and clinical variables. Specifically, we compared the groups on a diagnosis of autism, ID, ADHD, other NDCs, other psychiatric conditions, sex, and age. Little's MCAR test was significant (χ^2^ = 27.55, *p* < .001), which indicates that data were not missing completely at random. Those missing data on ABAS had a higher frequency of autism (χ^2^ = 6.70, *p* = .010), ID (χ^2^ = 6.27, *p* = .012), other NDCs (χ^2^ = 16.50, *p* < .001), and other psychiatric conditions (χ^2^ = 9.87, *p* = .002), but did not differ on ADHD, sex, or age.

The final sample consisted of 314 twin participants (163 MZ and 143 dizygotic [DZ] individuals, including a trio of DZ triplets and 8 individuals of unknown zygosity). In total, 101 participants were diagnosed with ADHD, 80 with autism, and 19 with ID. Furthermore, 59 had other NDCs (i.e. specific learning, tics, and speech/language disorders), and 67 had other psychiatric conditions (mostly affective disorders such as depression and anxiety disorders). Of those 142 participants with an NDC (i.e. ADHD, autism, or ID), 40 participants had two NDCs (33 with autism and ADHD, 5 with autism and ID, and 2 with ADHD and ID), while nine had been diagnosed with all three NDCs in question (i.e. autism, ADHD, and ID) and were included in more than one of the above categories. Sample characteristics are presented in Table 1.

**Table 1:** Study sample characteristics

	Total sample (314 individuals)	DZ twins (143 individuals)	MZ twins (163 individuals)
Sex (females), *n* (%)	126 (40.1%)	59 (41.3%)	62 (38.0%)
Years of age, mean/range	13.89/8–21	13.73/8–21	14.18/8–20
ADHD, *n* (%)	101 (32.2%)	61 (42.7%)	39 (23.9%)
Discordant pairs, *n*	50	37	12
Concordant pairs, *n*	24	11	13
Autism, *n* (%)	80 (25.5%)	37 (25.9%)	41 (25.2%)
Discordant pairs, *n*	48	27	19
Concordant pairs, *n*	16	5	11
ID, *n* (%)	19 (6%)	4 (2.8%)	14 (8.6%)
Discordant pairs, *n*	11	4	6
Concordant pairs, *n*	4		4
ABAS GAC, *M* (*SD*)	84.24 (23.06)	81.00 (23.47)	86.80 (22.41)

ABAS, adaptive behavior assessment system; ADHD, attention‐deficit/hyperactivity disorder; GAC, general adaptive composite; ID, intellectual disability.

### Diagnostic assessment

Participants were examined by experienced clinicians using an extensive battery of assessments during a 2½‐day visit at a clinical research unit as part of the RATSS protocol (Bölte et al., 2014). The diagnostic process is described in more detail in Appendix S1. DSM‐5 clinical consensus diagnoses of NDCs and other psychiatric conditions were determined by a group of clinicians using cognitive testing, clinical interviews, reports on medical history, and self‐ and parent‐report questionnaires. More specifically, the diagnosis of autism was informed by gold‐standard autism assessment instruments such as the Autism Diagnostic Observation Schedule Second Edition (ADOS‐2) with the participants, the Autism Diagnostic Interview‐Revised (ADI‐R) with the parent, as well as parental ratings with the Social Responsiveness Scale Second Edition (SRS‐2). The diagnosis of ADHD was supported by interviews with the parent using the Kiddie Schedule for Affective Disorders and Schizophrenia (K‐SADS) or the participant using the Structured Clinical Interview for DSM‐IV, depending on the participant's age, as well as interviews with the participant or the parent with the Diagnostic Interview for ADHD in adults (Kooij, 2010), and self and parental ratings using the Achenbach scales. A diagnosis of ID was determined using the Wechsler Intelligence Scales for Children or Adults or a combination of the Peabody Picture Vocabulary Test and Leiter scales.

### Adaptive functioning

Adaptive functioning was assessed using the Swedish version of the Adaptive Behavior Assessment System‐Second Edition (ABAS‐II) (Oakland & Harrison, 2008). The ABAS‐II is a well‐established instrument that exists in different forms, and for this study, the questionnaire for parental assessment was used. This parent‐rated instrument assesses adaptive behavior in children (5–21 years) in three domains: conceptual (communication, academic, self‐direction), social (leisure and social skills), and practical (community use, home living, health and safety, self‐care), as well as providing an overall General Adaptive Composite (GAC) combining domain results. The respondent answers 184 items on a Likert scale from 0 (= not able to perform the task), 1 (= never or almost never performs the task), 2 (= performs the task sometimes), or 3 (= always or almost always performs the task), where high scores indicate better functioning. By tabulating totals from the different skill areas, a raw score is calculated, which can be converted into normative standard scores for domains and the GAC (*M* = 100, *SD* = 15). ABAS‐II has shown adequate internal consistency, test–retest and inter‐rater reliability, and good content validity (Floyd et al., 2015). The Swedish version of ABAS‐II is standardized for the Swedish population and has well‐established psychometric properties with excellent internal consistency (Oakland & Harrison, 2008). In the present study, McDonald's Omega varied between .92 and .96.

### Statistical analyses

Analyses were performed using SPSS v28 and the ‘drgee’ package (v1.1.10) in RStudio (v2024.04.2) for conditional linear regressions within the generalized estimating equations (GEE). Sex differences in adaptive function (GAC and the three domains) were analyzed using independent sample *t*‐tests and differences in adaptive functioning by number of NDCs with one‐way ANOVAs with Tukey HSD post‐hoc tests. For the linear regressions, the exposure variables were defined as dichotomized categorical variables (e.g. diagnosis or no diagnosis of ADHD, autism, and ID; female sex or not; other psychiatric conditions; and other NDC), except for age, which was a continuous variable. The outcome variables, adaptive function defined as GAC or the three domains, were defined as continuous variables. The GEE framework does not make any distributional assumptions (e.g. normal distribution) and includes calculations of doubly robust standard errors (Goetgeluk & Vansteelandt, 2008; Zetterqvist & Sjölander, 2015). GAC and the three domains had a negatively skewed distribution (skewness ratio = −0.04 to −2.43) and a somewhat heavy‐tailed kurtosis (kurtosis ratio = 3.36 to 4.58). The linear regressions were executed in three steps. First, adaptive functioning was regressed on ADHD, autism, ID, other psychiatric conditions, other NDC, age, and sex in a fully adjusted model (including all independent variables) across the whole sample, where the twins were treated as separate individuals. In this model, we also tested interactions of age and sex by different forms of NDC on adaptive functioning as separate models. If an interaction was found in GAC, we repeated the model in the three domains. In the second step, within‐pair analyses were conducted in the adjusted model, assessing if the difference in exposure variable was correlated to differences in outcome within the pairs. In the within‐pair analyses, all variables shared within twin pairs, including shared environment (e.g. socioeconomic background, parenting styles) and genes (on average 50% of genes in DZ‐pairs and 100% of genes in MZ‐pairs), are implicitly controlled for. Thus, the within‐pair models allow us to study the potential influence of genetics and shared environment on the association between exposure and outcome. Only same‐sex twin pairs were included, so no adjustments for sex or age were necessary. In our last step, we recalculated the within‐pair analyses in the MZ sub‐cohort (here, DZ twins and those with unknown zygosity were excluded), fully adjusting for all shared genetics. Information on the GEE framework and the twin statistics is presented in Appendix S2. We adjusted for multiple comparisons using Bonferroni correction in the main GEE analyses, resulting in a *p*‐value of *p* < .006 being considered significant.

## Results

### Ratings on adaptive functioning

Descriptive statistics of adaptive functioning, stratified by diagnosis and sex, are presented in Table 2. Overall, those with NDCs had a GAC in the extreme‐low range, where especially those with ID had low scores. Scores for typically developing participants were in the average range. There were no sex differences in adaptive functioning across the sample (*t* = 0.028–1.738, *p* > .086).

**Table 2:** Descriptive statistics (mean value and standard deviation) over the distribution of adaptive functions across the entire sample and by sex

		Conceptual	Social	Practical	General adaptive composite
		*M* (*SD*)	*M* (*SD*)	*M* (*SD*)	*M* (*SD*)
ADHD	Female	60.97 (18.05)	68.03 (19.77)	75.61 (19.30)	67.15 (19.41)
	Male	64.75 (20.20)	69.03 (17.01)	74.18 (21.31)	68.03 (20.26)
	All	63.51 (19.52)	68.70 (17.86)	74.64 (20.59)	67.74 (19.89)
Autism	Female	58.04 (16.61)	57.00 (11.05)	70.00 (16.90)	60.35 (14.87)
	Male	63.46 (22.35)	62.56 (14.36)	68.15 (20.10)	62.83 (19.60)
	All	61.70 (20.71)	60.75 (13.56)	68.75 (19.03)	62.03 (18.15)
Intellectual disability	Female	47.50 (10.14)	53.63 (7.13)	64.25 (16.82)	53.00 (13.38)
	Male	51.09 (16.94)	59.55 (17.28)	60.82 (23.66)	54.27 (21.47)
	All	49.58 (14.24)	57.05 (13.95)	62.26 (20.59)	53.74 (18.10)
Typically developing	Female	97.20 (16.50)	94.26 (18.59)	102.51 (15.98)	99.51 (17.01)
	Male	96.97 (16.20)	93.20 (14.92)	100.33 (16.78)	98.33 (16.45)
	All	97.07 (16.27)	93.68 (16.61)	101.31 (16.40)	98.86 (16.65)

ABAS, adaptive behavior assessment system; ADHD = attention‐deficit/hyperactivity disorder.

### Ratings on adaptive functioning according to number of diagnoses

**Figure 1:**
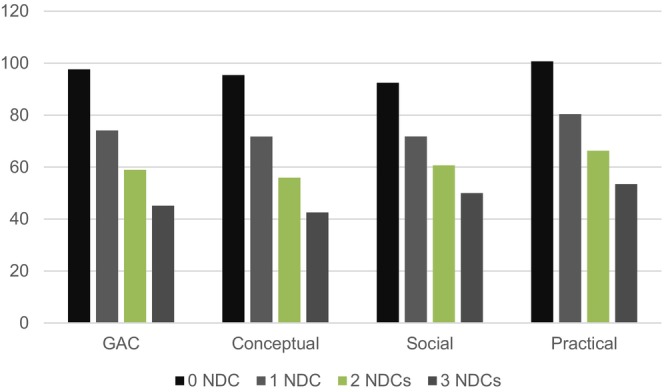
Parent‐rated adaptive functioning (mean) by numbers of NDCs. GAC, general adaptive composite

Mean levels for GAC and the three adaptive function domains (social, conceptual, practical) by the number of NDCs are presented in Figure 1. Those with an increasing number of NDCs had lower ratings on adaptive functioning for GAC (*F* = 95.37; *p* < .001). For the three specific domains, conceptual (*F* = 90.29; *p* < .001), social (*F* = 68.85; *p* < .001), and practical (*F* = 73.02; *p* < .001), those with more NDCs had lower ratings than those with fewer NDCs, except for those with two or three diagnoses, where no difference in adaptive functioning was found between these groups.

### Across and within‐pairs associations between NDCs and adaptive functioning

As shown in Table 3, all forms of NDCs were associated with lower scores on the GAC, with estimates being similar both across and within pairs. The confidence intervals of the estimates overlapped between the diagnoses, indicating a similar relationship. In contrast, in the within‐pair analysis in the MZ sub‐sample, the association between ADHD and adaptive functioning was attenuated and lost. Neither sex nor age was shown to have an impact across the sample. Although one interaction effect between autism and age was found (*b* = −2.01, CI = −3.27, −0.75; *p* = .002), indicating that for autistic individuals, higher age was related to lower adaptive functioning, which was not found in non‐autistic individuals. This interaction effect was limited to the social (*b* = −1.62, CI = −2.65, −0.58; *p* = .002) and the practical (*b* = −2.11, CI = −3.42, −0.80; *p* = .002) domains, indicating that autistic individuals had lower ratings of adaptive functioning in these domains with increasing age compared to non‐autistic individuals. ADHD, autism, and ID were also associated with lower scores in conceptual, social, and practical domains both across the sample and within pairs (Table S1). As with the GAC, the associations between ADHD and all three domains were lost within the MZ pairs.

**Table 3:** Across and within‐pairs associations with general adaptive composite across the entire sample and in MZ sub‐cohort

	Model 1 (across) 314 individuals		Model 2 (within) 309^a^ individuals		Model 3 (within MZ) 162^a^, ^b^ individuals	
	*b*	95% CI	*b*	95% CI	*b*	95% CI
ADHD	−14.87***	−19.22, −10.52	−16.19***	−22.21, −10.17	−0.81	−9.21, 7.59
Autism	−21.37***	−26.01, −16.74	−15.73***	−21.57, −9.90	−16.82***	−26.36, −7.28
Intellectual disability	−18.23***	−24.53, −11.92	−18.57***	−29.30, −7.84	−14.79***	−23.41, −6.17
Other psychiatric disorders	−8.21***	−12.75, −3.67	−10.07***	−15.46, −4.67	−1.71	−7.79, 4.36
Other NDC	−6.28*	−11.29, −1.27	2.55	−4.11, 9.22	2.69	−5.18, 10.56
Sex (female)	−0.15	−4.56, 4.25				
Age	0.21	−0.44, 0.86				

ADHD, attention‐deficit/hyperactivity disorder; NDC, neurodevelopmental conditions.

^a^ Only complete twin pairs/triplets included.

^b^ DZ twins and those with unknown zygosity were excluded; Bold indicates *p* < .006 (corrected for multiple comparisons).

*p < .05; **p < .01; ***p < .001.

## Discussion

This is the first study that has applied a co‐twin control design to investigate the associations between NDCs and adaptive function in a sample of carefully phenotyped twin pairs enriched for NDCs. We found that ADHD, autism, and ID were associated with lower parent‐rated scores on adaptive functioning, even when adjusting for co‐occurring mental health conditions, genetics, and shared environmental factors. However, when only including MZ pairs, the association with adaptive functioning attenuated and was lost for ADHD, indicating a genetic confounding. Age was found to moderate the association between autism and adaptive functioning, where older age and autism were associated with more challenges in adaptive functioning. We also found that an increasing number of NDCs were associated with more adaptive functioning challenges.

In line with our hypothesis, all forms of NDCs were associated with challenges in adaptive functioning. As an impact on adaptive functioning is a diagnostic criterion for NDCs (APA, 2013), the results were expected, although not trivial, as the criteria of impairment in the diagnostic manuals are not well defined, and this study used a comprehensive and standardized measure encompassing several domains of adaptive functioning. The associations also remained when adjusting for a wide range of co‐occurring conditions. It is important to acknowledge the existence of co‐occurring conditions, as these are common in NDCs (Antshel & Russo, 2019; Mutluer et al., 2022). The ABAS‐II scores for NDCs were in the lower to extreme‐low range of adaptive functioning. These levels of adaptive functioning are consistent with previous research on children diagnosed with autism (Laghi et al., 2022), ID (McKenzie et al., 2019), and ADHD (Lindblad et al., 2013), although our scores were lower compared to those from a study with adult females with ADHD (Kopp et al., 2023) and in a study on children and youth with ADHD (Krakowski et al., 2020). ADHD was foremost associated with challenges in conceptual skills, but also in social and practical skills. Academic function is also an area of concern for adolescents with ADHD (Öster, Ramklint, Meyer, & Isaksson, 2020). Autism was foremost associated with lower adaptive social functioning but almost equally low levels of conceptual and practical adaptive functioning. While social challenges are defining diagnostic features of autism, there is a clear need to also assess other areas of adaptive functioning to capture common challenges in everyday life in the group. ID was especially associated with lower conceptual skills, followed by practical skills and social skills, consistent with earlier research stating all three areas are impacted (Patel et al., 2020).

The association between autism, ID, and adaptive functioning remained in the within‐pair analyses, including in the MZ sub‐cohort where all shared genetics and environment are implicitly adjusted for. These findings indicate a non‐shared environmental contribution to the association between autism, ID, and adaptive functioning since this is the only factor that can make the pairs differ. This non‐shared factor could be due to other factors that may differ between the twins, for example birthweight and health conditions in early childhood (Isaksson et al., 2022). Interestingly, the association between ADHD and adaptive functioning was lost in the MZ subsample. Previous research suggests that especially autism, and to some degree also IQ and ID, are more strongly associated with perinatal and early postnatal risk loads compared to ADHD, indicating a role for non‐shared factors in autism and ID (Isaksson et al., 2022). In contrast, our finding does not provide support for non‐shared contributions in the link between ADHD and adaptive functioning, but rather indicates that the association is attributed to genetic factors that underlie both ADHD and adaptive functioning. As no previous research has applied a co‐twin control design when assessing the association between ADHD and adaptive functioning, our finding needs to be replicated. However, the result is in line with another study reporting a large and significant genetic overlap between ADHD and functional impairment using ACE modeling (Aydin et al., 2022).

Previous research (Ashwood et al., 2015; Liu et al., 2021; Sikora et al., 2012; Ward et al., 2022) has suggested a negative association between the co‐occurrence of NDCs and adaptive functioning. The co‐occurrence of NDCs has, for instance, been linked with increased school absenteeism and societal exclusion, unemployment, and educational attainment (Fleming et al., 2020). In line with this research and our hypothesis, our result suggests a cumulative effect regarding the relationship between the number of diagnoses and challenges in adaptive functioning. In the context of previous research indicating that co‐occurrence is common (Antshel & Russo, 2019; Mutluer et al., 2022), our findings are clinically relevant in supporting that assessments of NDCs should be conducted broadly.

Sex was not found to moderate the associations between different NDCs and adaptive functioning. Findings from previous literature are mixed, with reports of both no moderating effect of sex in autistic individuals (Hodge et al., 2021) and reports of autistic girls having lower daily living skills in parent reports despite having similar overall performance on gold‐standard diagnostic measures (Ratto et al., 2018). Similarly, for ADHD, a moderating effect in children with ADHD has been found, where girls with ADHD scored higher on social adaptive functioning than boys across all ages (Mahendiran et al., 2019), whereas among adults, females more frequently reported self‐confidence issues, while males more often reported relationship or family‐related impairments (Platania, Starreveld, Wynchank, Beekman, & Kooij, 2025).

As to age, we had a relatively large age range in the sample, stretching from childhood to young adulthood, which needs to be considered when discussing findings in relation to adaptive functioning. There is an increased acquisition of adaptive skills during this developmental period, supporting an increased independence with age, and the association between age and adaptive functioning may not be linear. At the same time, age was adjusted for since the ABAS scores are converted into composite scores based on the participants' age, and in the within‐pair models, the twins have the same age. Despite this, we did find an interaction effect of age on adaptive functioning in autistic and non‐autistic individuals across the pairs, where older age was associated with more challenges in adaptive functioning in autistic individuals. Similarly, within a large European multicenter study that used another common tool for measuring adaptive behavior, the Vineland Adaptive Behavior Scales, older age was negatively associated with adaptive functioning in autistic children and adults (Tillmann et al., 2019). Autistic individuals may not develop adaptive functioning skills at the same rate as their non‐autistic peers, resulting in an increasing discrepancy with age. Additionally, the demands and complexity of social environments often increase with age, placing additional pressure on adaptive functioning skills, and our results may reflect that autistic individuals often struggle with transitioning from adolescence to young adulthood and the demands of independence (Volkmar, Jackson, & Hart, 2017). In contrast, symptoms of ADHD have been suggested to attenuate with increasing age (Vos & Hartman, 2022), where especially symptoms of hyperactivity and impulsivity are reduced in adolescence and adulthood, while symptoms of inattention remain (Faraone et al., 2021). However, we observed no improvement in adaptive functioning with age, also suggesting that symptoms and function do not always correspond, although consideration must be given that our oldest participant was only 21 years old. At the same time, others have reported that impulsive symptoms become more central and important during adolescence, with verbal impulsivity being particularly important during adulthood (Martel, Levinson, Langer, & Nigg, 2016).

While the present study acknowledges the importance of conducting broad assessments of adaptive functioning in the presence of NDCs, measures of adaptive functioning are limited by predominantly taking an impairment‐orientated approach to functioning (D'Arcy et al., 2022). But functioning itself, as defined by the World Health Organization International Classification of Functioning, Disability, and Health (ICF), is neutral referring to both functioning strengths and challenges, and should be regarded as resulting from the dynamic interaction between body functions/structures, activities, and participation within an environmental context (Bölte et al., 2024a, 2024b). As such, adaptive functioning challenges observed in NDC populations may be influenced by environmental considerations and a lack of ‘fit’ between an individual, their activities, and their environments. For instance, among autistic individuals, research has begun to frame social communication challenges as a problem arising between two people (a double‐empathy problem) as opposed to a challenge arising solely from the autistic individual (Milton, Gurbuz, & López, 2022). Indeed, the fact that we observe that adaptive functioning in both autism and ID cannot be explained by genetic or shared environmental factors adds credence to the notion that a range of environmental and other contextual factors, such as peers and schooling, may be influencing the functioning of these individuals. Hence, future studies should include measures that also capture environmental and contextual factors that may act to support or hinder functioning. Such investigation may benefit from employing tools such as the ICF Core‐Sets for Autism and ADHD (Bölte et al., 2024a, 2024b), which are rigorously developed to capture all factors influencing functioning in these conditions, which may better reflect the lived experience of individuals with NDCs that can inform support provision (Bölte, 2023).

To fully evaluate the validity of our findings, some limitations of the current study must be considered. We used parental ratings when assessing adaptive functioning. These ratings may be biased in several ways, including confirmation or recall bias. For instance, MZ pairs may correlate more on parental ratings given their similarity (Saudino, Cherny, & Plomin, 2000), resulting in an attenuation of the estimates. Even though we had a relatively large population of twins carefully phenotyped for NDCs, statistical power could have been reduced by including several study variables and stratifying the sample into subsamples. For instance, only 19 participants had ID, and for the MZ subsample, 12 pairs were discordant for ADHD. Further, some participants had missing data on ABAS. Since these were relatively few (5%), we analyzed the missing data and performed a complete‐case analysis as suggested by previous literature (Cummings, 2013). Although we compare adaptive functioning between different NDCs, the exposure variable (presence of NDC diagnosis) and the outcome variable (adaptive function) are closely linked, given that functional impairment is included in the diagnostic criteria for all NDCs; this will affect the estimates. There are also some differences in the wording of functional impairment between the conditions in the DSM‐5. For ADHD, the symptoms should interfere with or reduce the quality of social, academic, or occupational functioning; for autism, the symptoms should cause significant impairment in social, occupational, or other important areas of functioning; while for ID, adaptive functioning is more explicitly mentioned with a failure to meet developmental and sociocultural standards, limiting functioning in areas such as communication, social participation, and independent living. Even though we used a broad measure of adaptive functioning, capturing several areas of functioning, the domains included in ABAS‐II were initially selected according to guidelines of the American Association of Intellectual Disabilities and may hence be more relevant for ID. At the same time, it is clearly stated that the instrument may also be used to assess adaptive behavior in individuals with other impairments, including autism or ADHD (Floyd et al., 2015; Gray & Carter, 2013), with their core symptoms corresponding to the skill areas targeted in ABAS.

We did not separate between the different presentations of ADHD, including the inattentive, the hyperactive, and the combined type, which might have influenced the results. For instance, it has been reported that only those with the predominantly inattentive presentation have challenges in adaptive functioning (Stavro, Ettenhofer, & Nigg, 2007). Age was already indirectly adjusted for, given that the scores are converted into composite scores based on the participants' age, which could make the adjustment of age redundant. At the same time, however, the association between NDC and adaptive functioning varies with age and may still be of importance, as NDC may affect adaptive functioning differently over development. Lastly, our sample is biased toward twins discordant for NDCs. Although this recruitment strategy makes it inappropriate to use bivariate twin models such as ACE models, it is appropriate for investigating the association between NDCs and associated features while controlling for familial confounders. On balance, the strengths of the study include a relatively large and rare sample of well‐phenotyped MZ and DZ twins, including a considerable number of twins in Sweden concordant and discordant for NDCs (Lichtenstein, Carlström, Råstam, Gillberg, & Anckarsäter, 2010).

## Conclusions

In conclusion, ADHD, autism, and ID were associated with increasing challenges in adaptive functioning, both across individuals and within co‐twin pairs, indicating that these NDCs have a unique impact on adaptive functioning despite their high co‐occurrence. The co‐twin control analyses in the MZ subset indicate a genetic confounding on the association between ADHD and adaptive functioning, suggesting that shared genetic factors may explain the link between ADHD and adaptive functioning, influencing both variables. This finding needs to be replicated, and if confirmed, could indicate that siblings at elevated familial likelihood for ADHD are in need of support even if they do not fulfill the diagnostic criteria for ADHD. Further, screening tools and assessments should include adaptive skills, and interventions should more directly target adaptive functioning and not just ADHD symptoms. On the contrary, for autism and ID, genetics and shared environment did not explain the level of adaptive functioning, which is why future research should include non‐shared environmental factors associated with autism and ID, such as low birthweight (Isaksson et al., 2022), but also factors capturing the environmental context that may support or hinder functioning for the individual, such as skill‐building programs and environmental accommodations and modifications. Our findings also indicate that the presence of multiple NDCs is associated with more challenges in adaptive functioning than the presence of a single diagnosis. This indicates that multiply diagnosed individuals have higher support needs and stresses the importance of screening for co‐occurring NDCs and increased community services for people with more complex neurodivergent phenotypes. Especially autistic individuals may face increased challenges with increased age, emphasizing the potential need for ongoing support for more individuals in this group.

## Ethical considerations

The study was approved by the Swedish Ethical Review Authority (Dnr. 2016/1452‐31, date: 2017‐02‐10), and written and oral informed consent was obtained from all participants.

**Key points**

**What's known?**

- Neurodevelopmental conditions (NDCs) such as autism, ADHD, and ID are associated with challenges in adaptive functioning.
- Research on the influence of co‐occurring conditions and familial factors on the association between NDCs and adaptive functioning is limited.

**What's new?**

- ADHD, autism, and ID were all uniquely associated with challenges in adaptive functioning, where the presence of multiple NDCs leads to more challenges.
- Increasing age was associated with more challenges in adaptive functioning among autistic individuals, emphasizing the need for ongoing support.

**What's relevant?**

- Findings among monozygotic twins indicate a genetic confounding of the association between ADHD and adaptive functioning.

## Supporting information


**Appendix S1.** Information about RATSS diagnostic procedure.
**Appendix S2.** Information about the statistical models.
**Table S1.** Across and within‐pairs associations with conceptual, social and practical skills across the entire sample and in MZ sub‐cohort.

## Data Availability

The datasets are not readily available because of regulations in the ethical approval, requiring among others a data sharing agreement. Requests to access the datasets should be directed to sven.bolte@ki.se.
