# Supplementary material for: Neurodevelopmental conditions and adaptive functioning – a co‐twin control study
Source: J Child Psychol Psychiatry. 2025 Nov 4;67(5):777–87. doi: 10.1111/jcpp.70073 (PMC13102047; doi:10.1111/jcpp.70073)
Supplement: Supplementary file 1 — Appendix S1. Information about RATSS diagnostic procedure. Appendix S2. Information about the statistical models. Table S1. Across and within‐pairs associations with conceptual, social and practical skills across the entire sample and in MZ sub‐cohort. [file JCPP-67-777-s001.docx]

**Neurodevelopmental conditions and adaptive functioning –**

**a co-twin control study**

**Supporting Information**

**Appendix S1.** Information about RATSS diagnostic procedure

The RATSS assessments were conducted during a 2½ day visit at the research clinic of Karolinska Institutet Center for Neurodevelopmental Disorders (KIND) in Stockholm by well-trained nurses, psychologists, and physicians. DSM-5 clinical consensus diagnoses of NDCs and other psychiatric conditions were determined by a group of clinicians based on all available data.

Assessments in RATSS include, among others, first choice standardized diagnostic tools for Neurodevelopmental conditions (NDCs), such as the Autism Diagnostic Interview–Revised (ADI-R) and Autism Diagnostic Observation Schedule, Second Edition (ADOS-2) for autism, and the Diagnostic Interview for ADHD in Adults (DIVA) for ADHD. Quantitative measures administered included the Social Responsiveness Scale, Second Edition (SRS-2) and Autism-Spectrum Quotient (AQ) for capturing autistic-like traits, and the Camouflaging Autistic Traits Questionnaire CAT-Q for evaluating camouflaging behaviours. For ADHD, Conners 3, the Adult ADHD Self-Report Scale (ASRS), and Achenbach Scales for ADHD were administered. Parent- and self-report on Achenbach Scales were also used to measure general psychopathology, and clinical interviews (Kiddie Schedule for Affective Disorders and Schizophrenia [K-SADS] or Structured Clinical Interview for DSM Disorders [SCID]) informed psychiatric assessments. Assessment for general abilities and neuropsychology included the Wechsler Intelligence Scale for Children, Fourth Edition (WISC-IV) or the Wechsler Adult Intelligence Scale, Fourth Edition (WAIS-IV), Leiter scales, and the Peabody Picture Vocabulary Test (PPVT). Social cognition was captured via the Reading the Mind in the Eyes and Movie for the Assessment of Social Cognition (MASC). Visual perception was evaluated via the Fragmented Picture Test and Embedded Figures Test. Executive functioning was assessed via the Tower Test (from D-KEFS), the Wisconsin Card Sorting Test (WCST), and the Trail-Making Test A & B. Communication was evaluated using the Children’s Communication Checklist/Children’s Communication Checklist–Adult (CCC/CC-A), and sensory processing via the Sensory Profile. Adaptive functioning was measured using the Adaptive Behavior Assessment System, Second Edition (ABAS-II) and the WHODAS 2.0. We also captured quality of life using the European Health Interview Survey–Quality of Life (EUROHIS-QOL). For some informative twins, an online synesthesia screening is applied. Medical and family history and socio-demographic information were also collected. In addition, head circumference, weight, height, and blood pressure were measured.

**Appendix S2.** Information about the statistical models

Regression models were fitted using the conditional generalized estimating equations (GEE) framework that accounts for the use of related individuals in the analysis, including calculation of robust standard errors. The model is not limited to distributional assumption (e.g., normal distribution or such) of outcomes and residuals, and the GEE is a recommended analytic approach in co-twin control designs and appropriate for continuous as well as binary outcomes (Scurrah & Hopper, 2019). Here we used continuous outcome (i.e., ABAS composite scores), identity link within the drgee function in R was used. The conditional GEE is described in Goetgeluk & Vansteelandt article (2008).

In the across-pair analyses, with linear regression models for estimates of associations between NDCs and adaptive functioning (between-pairs estimates), twins were treated as individuals/singletons, although clustered standard errors were used accounting for the twin correlation. In these analyses, adjustment was made for sex and age.

In the within-pair analyses, conditional linear regression model was used for estimates of association within-pairs (the difference in the exposure variable within a pair is correlated to the difference in the outcome variable within the same pair) after adjusting for factors shared within twins. The within-pair analyses implicitly control for everything shared by the twins within a pair (e.g., shared environment, including family socioeconomic status and parenting styles, as well as on average 50% of segregating genes in DZ-pairs and 100% of genes in MZ-pairs), which is kept constant in the model and do not have an effect on the outcome. There is no need to adjust for gender and age.

The within-pair analyses were further calculated in the MZ-subset to allow us to further decipher the complexity of genotype-environment-phenotype associations since this allow us to control for all genetics. The within-pair associations among MZ twin-pairs offers unique opportunities to investigate possible pathways focusing on the within-twin pair differences with respect to unique experiences, i.e., nonshared factors.

**References**

Goetgeluk S, Vansteelandt S. Conditional generalized estimating equations for the analysis of

clustered and longitudinal data. Biometrics 2008;64(3):772-80.

Scurrah KJ, Hopper JL. Twin research: Designs and analytic approaches. Conversations in

Twins Research. Twins Research Australia. 2019.

<https://www.twins.org.au/research/tools-and-resources/125-conversation-in-twin-research/377-twin-research-designs-and-analytic-approaches>

**Table S1.** Across and within-pairs associations with conceptual, social and practical skills across the entire sample and in MZ sub-cohort

|  | | Model 1 (across) N=314 individuals | | Model 2 (within) N=309^a^ individuals | | Model 3 (within MZ) N=162^a,b^ individuals | |
| --- | --- | --- | --- | --- | --- | --- | --- |
|  |  | b | 95% CI | b | 95% CI | b | 95% CI |
| ADHD | Conceptual | **-18.63***** | -23.24, -14.02 | **-20.04***** | -26.46, -13.61 | -2.97 | -11.69, 5.75 |
|  | Social | **-10.33***** | -14.52, -6.14 | **-11.39***** | -17.53, -5.26 | -1.24 | -11.91, 9.43 |
|  | Practical | **-12.04***** | -16.64, -7.44 | **-13.06***** | -19.13, -6.99 | 0.84 | -6.57, 8.26 |
| Autism | Conceptual | **-17.39***** | -22.51, -12.26 | **-12.41***** | -19.22, -5.60 | **-15.55**** | -25.52, -5.58 |
|  | Social | **-21.63***** | -25.85, -17.41 | **-17.29***** | -23.06, -11.53 | **-15.52***** | -23.76, -7.28 |
|  | Practical | **-19.62***** | -24.41, -14.84 | **-13.86***** | -19.64, -8.07 | **-15.12**** | -24.74, -5.50 |
| Intellectual disability | Conceptual | **-20.89***** | -27.11, -14.67 | **-22.87***** | -33.97, -11.78 | **-19.50***** | -30.27, -8.72 |
|  | Social | **-11.85***** | -17.10, -6.60 | -13.48** | -23.61, -3.35 | -13.25* | -25.14, -1.36 |
|  | Practical | **-16.13***** | -23.18, -9.07 | **-14.94**** | -24.90, -4.98 | **-9.91**** | -16.38, -3.43 |
| Other psychiatric disorders | Conceptual | **-7.31**** | -11.97, -2.62 | **-8.74**** | -14.09, -3.39 | -1.70 | -8.48, 5.08 |
|  | Social | **-8.24***** | -12.69, -3.79 | -7.66** | -13.26, -2.06 | -2.50 | -8.96, 3.97 |
|  | Practical | **-7.09**** | -11.89, -2.29 | **-9.76***** | -15.37, -4.14 | -0.45 | -6.64, 5.74 |
| Other NDC | Conceptual | **-10.16***** | -15.35, -4.96 | -3.20 | -10.15, 3.76 | -1.33 | -8.28, 5.61 |
|  | Social | -2.73 | -7.70, 2.24 | 5.41 | -1.77, 12.59 | 5.00 | -5.38, 15.38 |
|  | Practical | -4.16 | -9.50, 1.17 | 4.27 | -2.89, 11.43 | 3.65 | -4.11, 11.40 |
| Female sex | Conceptual | -1.81 | -6.28, 2.66 |  | | | |
|  | Social | -0.46 | -4.88, 3.96 |  |  |  |  |
|  | Practical | 1.48 | -2.90, 5.85 |  |  |  |  |
| Age | Conceptual | -0.29 | -0.29, 0.36 |  |  |  |  |
|  | Social | -0.05 | -0.64, 0.55 |  |  |  |  |
|  | Practical | 0.63 | -0.03, 1.30 |  |  |  |  |

*Note.* *p < 0.05; **p<0.01, ***p < 0.001; ^a^ Only complete twin pairs included. ^b^ DZ twins and those with unknown zygosity were excluded. Bold indicate p < 0.006 (corrected for multiple comparisons)
